# 4-Phenylbutyrate Rescue in *GABRA1* Variants Associated with Developmental Epileptic Encephalopathies: From Cell and Mouse Models to Humans

**DOI:** 10.3390/cells15151327

**Published:** 2026-07-24

**Authors:** Ziang (Debbie) Song, Kirill Zavalin, Wangzhen Shen, Melissa B. DeLeeuw, Genevieve X. Hunn, Ria S. Eda, Li Ma, Juexin Wang, Jing-Qiong Kang

**Affiliations:** 1 Department of Neurology, Vanderbilt University Medical Center, Nashville, TN 37232, USA; ziang.song.1@vumc.org (Z.S.); kirill.zavalin@vanderbilt.edu (K.Z.); wangzhen.shen@vumc.org (W.S.); genevieve.x.hunn@vanderbilt.edu (G.X.H.); ria.s.eda@vanderbilt.edu (R.S.E.); li.ma@vumc.org (L.M.); 2 Vanderbilt Brain Institute, Vanderbilt University, Nashville, TN 37232, USA; 3 Department of Biomedical Engineering and Informatics, Luddy School of Informatics, Computing, and Engineering, Indiana University Indianapolis, Indianapolis, IN 46202, USA; 4 Vanderbilt Kennedy Center, Vanderbilt University, Nashville, TN 37232, USA; 5 Department of Pharmacology, Vanderbilt University, Nashville, TN 37232, USA

**Keywords:** 4-phenylbutyrate (PBA), GABA_A_ receptors, Gabra1 A322D knockin mice, GABRA1, proteostasis, developmental and epileptic encephalopathy (DEE)

## Abstract

Disease variants in *GABR* genes encoding γ-aminobutyric acid type A receptor (GABA_A_R) subunits are major causes of developmental and epileptic encephalopathies (DEEs). There is no effective treatment for these DEEs, although the GABA_A_R is a major target for antiseizure drugs. We previously identified the therapeutic effect of 4-phenylbutyrate (PBA) in *Gabrg2*^+/Q390X^ knockin DEE mice and in this study tested the effect of the drug in *GABRA1* variants that encode the α1 subunit of GABA_A_R. We used a multidisciplinary approach including in silico structural modeling, flow cytometry, patch-clamp recordings and biochemistry in conjunction with differential tagging of the wildtype (WT) and the mutant alleles to evaluate the effect of PBA on rescue of GABA_A_R subunit expression, surface trafficking, and function in vitro in a heterologous HEK293T cell model and in vivo in *Gabra1*^+/A322D^ mice. We found that the α1 subunit expression at both the total level and the cell surface was reduced when the variant α1 protein was present, suggesting reduced functional receptor availability on the cell membrane and synapse. Patch-clamp recordings identified that α1 variants reduced GABA-evoked current amplitude. In silico prediction indicated reduced protein stability for *GABRA1* variants by negative ∆∆G values. PBA increased both total and surface expression of WT α1 and α1 variants and improved expression of both WT and variant α1 alleles when these were co-expressed. Importantly, PBA also increased the GABA_A_R expression in the cortex and thalamus of the *Gabra1*^+/A322D^ mice. This study indicates that PBA is a promising treatment option for DEEs associated with *GABRA1* mutations. Our previous work has demonstrated that PBA improves proteostasis by enhancing expression of the WT allele, repairing the mutant allele, and reducing endoplasmic reticulum stress in other DEEs associated with *GABRG2* and *SLC6A1* mutations. Importantly, it can mitigate seizures and improve neurobehavioral phenotypes at behavioral levels. Based on this and our previous work on *GABRG2* and *SLC6A1* mutations, we propose that PBA holds promise as a common medicine for multiple genetic neurologic disorders that share the proteostasis pathology with a broad clinical application in DEEs.

## 1. Introduction

Pathogenic variants in genes involved in inhibitory neurotransmission comprise a substantial fraction of known monogenetic causes of neurologic disorders, including epilepsies and more severe developmental and epileptic encephalopathies (DEEs) [1,2]. Among these, variants in *GABR* genes that encode subunits of γ-aminobutyric acid (GABA) type A receptors (GABA_A_Rs) are particularly prominent. GABA_A_Rs mediate most synaptic inhibition and a significant portion of tonic inhibition in the brain and are essential for controlling neuronal excitability. GABA_A_Rs are ionotropic ligand-gated chloride channels comprising five subunits with nineteen known isoforms. Most synaptic GABA_A_ Rs are comprised of two α1-3, two β2/3, and one γ2S subunit. The α1β2/3γ2S combination is by far the most prevalent in the brain, and the *GABRA1*, *GABRB2*, *GABRB3*, and *GABRG2* genes encoding these subunits are the most frequently occurring *GABR* pathogenic variants [3]. Most known cases are dominant *de novo* mutations, with a smaller proportion of familial cases [3].

In recent years, our lab has begun developing a promising new therapy for monogenetic epilepsies, including *GABR* variant-mediated disorders, using 4-phenylbutyrate (PBA), a small molecule with chemical chaperone and histone deacetylase inhibitor properties that is approved by the FDA for treatment of urea cycle disorders [4,5]. *GABR* mutations destabilize the subunit protein, impacting intracellular trafficking and/or interaction with partnering subunits, which affects GABA_A_R expression and functional properties [3,6,7,8]. PBA remedies this pathomechanism two-fold by stabilizing mutant protein in mild cases where this is possible or by assisting the clearance of severely misfolded proteins and improving expression of the functional wildtype (WT) allele and partnering subunits in a fashion similar to its effects in other monogenetic disorders [4,9,10,11,12,13,14,15,16,17,18] and acquired injuries [19,20]. Previously, we demonstrated this for the *GABRG2*(Q390X) variant associated with Dravet syndrome, which exhibits a severe trafficking pathology where mutant protein is retained in the endoplasmic reticulum and has a dominant negative effect on partnering subunits, significantly reducing GABA_A_R expression and synaptic inhibition and causing seizures [8]. Treatment with PBA promoted clearance of the mutant protein and improved expression of the functional WT allele and partnering subunits in cell and mouse models, improving synaptic inhibition and significantly reducing the seizure burden [21]. Additionally, we have been successful in using PBA to treat *SLC6A1* variant-mediated disorders in both preclinical models [22,23] and humans [24,25], which have a similar pathomechanism. For these disorders, mutant variants in GABA transporter 1 are frequently misfolded, resulting in reduced expression and transporter-mediated GABA uptake. Treatment with PBA improved intracellular trafficking and expression of the transporter, increased GABA uptake in cells and the *SLC6A1*(S295L) mouse model, and reduced the mouse seizure burden and neurobehavioral phenotypes [22,23,24,26,27]. These studies formed the basis for a clinical trial for PBA treatment, which reported seizure reduction or resolution in 70% of the *SLC6A1* patients tested [25].

**Figure 1:**
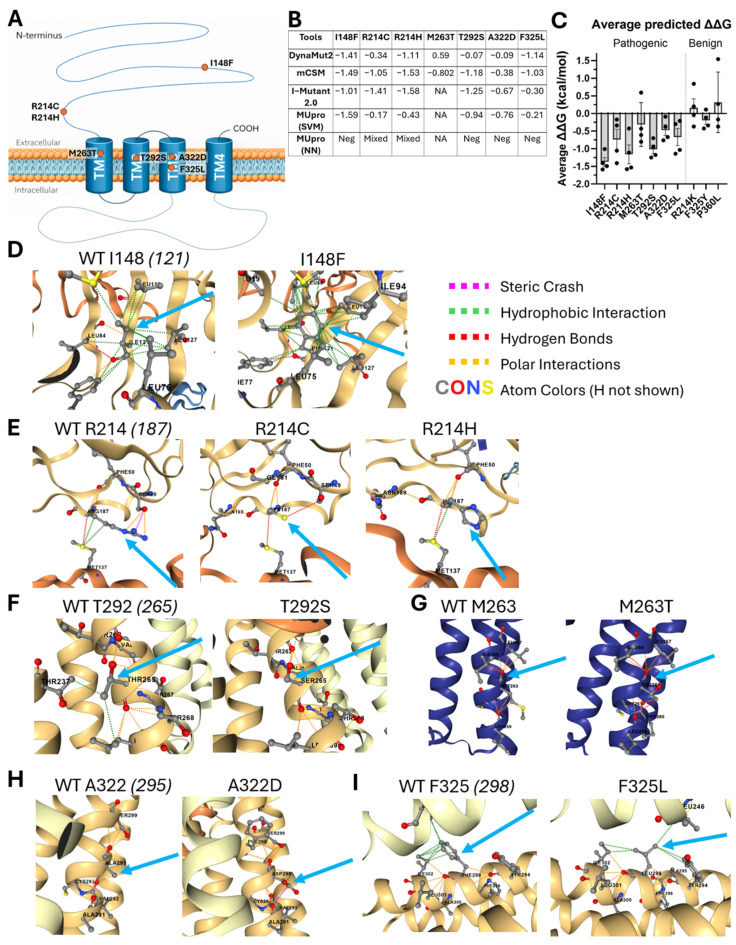
*GABRA1* variants disrupt local intermolecular interactions and destabilize the α1 subunit. (**A**) Locations of variants included in this study within the α1 subunit. (**B**,**C**) Predicted changes in protein stability (ΔΔG) for selected *GABRA1* variants calculated using multiple in silico prediction tools. The table in (**B**) shows scores generated by each prediction tool, and the bar graph in (**C**) shows the mean predicted ΔΔG for each variant. Disease-associated and benign variants are separated by the dashed line. (**D**–**I**) DynaMut2-predicted local environments around the variant residues based on the previously characterized GABA_A_R structure (PDB: 9CRS); chain D was used. For each variant, the WT structure is shown on the left and the predicted structural change due to the variant is shown on the right. The peptide length used in the PDB: 9CRS structure report was slightly shorter than that of the full-length human α1. For each residue, the first peptide number corresponds to the position within the full peptide and the second number corresponds to the peptide number within the structure. Abbreviations: Negative (Neg), Not Applicable (NA).

Clinical trials for *GABR* variant-mediated disorders are also under way, and a significant need exists for translational studies evaluating the efficacy of PBA for *GABR* mutations, including subunits that have not yet been evaluated, including *GABRA1*. In this study, we evaluate the effect that *GABRA1* mutations have on GABA_A_R subunit stability and expression and function. We also determine if PBA can restore these deficits. We focus on the *GABRA1* variants I148F, R214C, R214H, M253T, T292S, and F325L (Figure 1A; Table 1) obtained from the CURE GABA-A patient organization. Additionally, we include the *GABRA1*(A322D) variant associated with autosomal dominant juvenile myoclonic epilepsy. A322D was the first *GABRA1* variant to be identified, and it has been extensively characterized, displaying a misfolding/trafficking pathology with a modest dominant negative effect on partnering subunits [28,29,30,31,32]. Functional studies have also been done on R214C, R214H, T292S; R214C and R214H, which were identified in patients with Dravet syndrome and exhibit gating defects, showing diminished GABA_A_R currents, with differing reports regarding expression [33,34,35,36]. The T292S variant is associated with severe developmental delay without epilepsy and shows increased GABA sensitivity and normal expression, indicating a gain in GABA_A_R function [37]. No functional characterization has yet been done for I148F, F325L identified in a patient with epilepsy [38], and M253T identified in a West syndrome patient [39]. To predict the destabilizing effects these variants have on the subunit protein, we used computational tools and measured the expression of these variants along with the WT allele and partnering subunits in the HEK293 heterologous expression system and *Gabra1*^+/A322D^ mice. Then, we tested if PBA treatment can improve GABA_A_R subunit expression.

## 2. Materials and Methods

Mice

The heterozygous *Gabra1^+/A322D^* mouse line with the C57BL/6J background was kindly provided by Dr. Martin Gallagher of the Vanderbilt University Medical Center. This mouse line has previously been characterized, and genotyping was performed using Transnetyx using previously published probes [40]. The mice used in the study were males aged between 2 and 4 months old. The mice were housed in a temperature- and humidity-controlled environment with a 12 h light/dark schedule. Water and food were provided ad libitum. All animal procedures were performed in accordance with protocols approved by the Vanderbilt University Institutional Animal Care and Use Committee (IACUC). Animals were included based on confirmed genotype and normal health status. Animals with illness or unrelated injury were excluded. No collected animal data were excluded from the analysis.

GABA_A_R subunit cDNA and mutagenesis

Human α1, β2, and γ2S GABA_A_R subunit cDNAs were subcloned into the mammalian expression vector pcDNA3.1(+) as previously described [41,42]. An HA epitope tag was inserted into the 4th and 5th amino acids of the extracellular N-terminus of the WT α1 subunit of the mature peptide sequence. The HA tag was only added in the WT α1 construct to allow selective detection of the WT subunit protein in the heterozygous expression experiment. Site-directed mutations in the α1 subunit were generated using the Agilent QuikChange Site-Directed Mutagenesis Kit (Santa Clara, CA, USA). All mutations were verified by Sanger DNA sequencing. Mutant plasmid DNA for transfection was prepared using the QIAGEN MaxiPrep Kit (Venlo, the Netherlands).

HEK293T cell transfection

Transient transfection was performed using polyethylenimine (PEI) in human embryonic kidney 293T (HEK293T) cells. HEK293T cells were seeded in 60 mm dishes 24 h prior to transfection. For each 60 mm dish, 3 μg total plasmid DNA was transfected using PEI at a ratio of 2.5 μL PEI per 1 μg DNA. For homozygous receptor expression, α1, β2, and γ2S subunit cDNAs were transfected at a 1:1:1 ratio. For heterozygous α1 expression, equal amounts of wildtype and mutant α1 plasmids were co-transfected with β2 and γ2S cDNAs at a ratio of 0.5:0.5:1:1 for α1 (mutant or control):α1HA(WT):β2:γ2S. After 48 h of incubation, the cells were harvested and flow cytometry experiments on GABA_A_R expression were performed as previously described [31,43].

For electrophysiology experiments, HEK293T cells were transfected with a total of 3 μg plasmid DNA at a 1:1:0.5:0.3 ratio of α1:β2:γ2S:GFP using 1.5 μL Lipofectamine 2000 per 1 μg DNA, and cells were lifted gently using TrypLE (ThermoFisher Scientific, Waltham, MA, USA) to preserve optimal cellular membrane conditions for patch-clamp recordings. Transfected cells were identified based on the presence of GFP; typically, we observed a 10–20% transfection rate.

PBA administration in vitro and in vivo

The stock solution of PBA (Sigma-Aldrich, P21005, St. Louis, MO, USA) was prepared in dimethyl sulfoxide (DMSO). The optimal concentration (2 mmol·L^−1^) and treatment duration (24 h) for HEK293T cells were determined in a previous study and were used throughout this study.

For in vivo experiments, mice received either vehicle or PBA (20 mg/mL in 0.9% saline, pH 7.4) at 100 mg/kg/day for 7 d through daily intraperitoneal injections; this dose was determined based on our prior experiments in *Slc6a1*^+/S295L^ mice [22] and is comparable to the 12.4 g/m^2^/day dosing used in clinical trial patients [25]. Brain tissues were collected 24 h after the final PBA administration (day 8) for subsequent Western blot analysis. To minimize potential confounders, treated and untreated animals were derived from the same litters and treated with vehicle or PBA under identical conditions based on random assignment. Tissues were collected at the same time, and all samples were processed and analyzed in parallel. No formal a priori sample size calculation was performed; sample size was determined by the number of available mice.

Surface expression of α1 subunit evaluated by flow cytometry

The protocol was based on our earlier studies [41,43]. Cells were detached by trypsinization, washed, and resuspended in FACS buffer (phosphate-buffered saline supplemented with 2% fetal bovine serum and 0.05% sodium azide). For surface expression analysis, non-permeabilized cells were incubated with anti-GABA_A_R α1 antibody for 2 h and then incubated with fluorophore Alexa-647 conjugated goat anti-mouse secondary antibody (1:2000) for 1 h at 4 °C. Cells were subsequently washed and analyzed on a 3-laser LSR II flow cytometer (BD Biosciences, Franklin Lakes, NJ, USA) at the Vanderbilt Flow Cytometry Core. Cell populations were first gated based on forward and side scatter (FSC/SSC) to exclude debris. Approximately 10,000 events were collected per sample. Untransfected cells and isotype controls were included to define background fluorescence. Surface α1 expression was quantified as mean fluorescence intensity (MFI) using FlowJo v7.1 (Tree Star, Ashland, OR, USA).

Western blotting

The protocol was based on our earlier studies [44,45] and was similar for mouse samples and HEK293T cells. Live transfected HEK293T cells were washed three times with phosphate-buffered saline (PBS, 1×, pH 7.4); alternatively, mouse brain samples were dissected in PBS. Then, samples were lysed in RIPA buffer (20 mM Tris, 20 mM EGTA, 1 mM DTT, and 1 mM benzamidine) supplemented with protease inhibitors (0.01 mM PMSF, 0.005 μg/mL leupeptin, and 0.005 μg/mL pepstatin) for 30 min at 4 °C. Cell lysates were sonicated and clarified by centrifugation. Protein concentrations were determined using the Bradford assay. Equal amounts of protein were mixed with Laemmli sample buffer and separated by SDS-PAGE. Proteins were transferred onto PVDF membranes. Membranes were incubated overnight at 4 °C with primary antibodies. The primary antibodies used were mouse anti-GABA_A_R α1 1:500 (Millipore Sigma MABN489, Burlington, MA, USA), rabbit anti-GABA_A_R β2 1:500 (Millipore Sigma AB5561, Burlington, MA, USA), rabbit GABA_A_R β3 1:500 (Novus NB300-199, Centennial, CO, USA), rabbit anti-GABA_A_R γ2 1:500 (Synaptic Systems 224003; Gottingen, Germany), rabbit anti-HA 1:500 (Cell Signaling Technology 3724; Danvers, MA, USA), and anti-ATPase 1:1000 (Developmental Studies Hybridoma Bank a6F). The secondary antibodies used were LI-COR IRDye 680LT Goat anti-Mouse IgG Secondary Antibody (926-68020) and IRDye 800CW Goat anti-Rabbit IgG Secondary Antibody (926-32211), both 1:10,000. Band intensities were quantified using LiCor Image Studio software version 6. Uncropped full-length gels for Western blots were provided in the Supplementary Materials.

Electrophysiology

HEK293Ts were patched with 1.2–1.4 MΩ borosilicate glass electrodes (World Precision Instruments, Sarasota, FL, USA, TW150-4, pulled with Narishige PP-830 or Sutter P-2000 pullers) filled with an internal solution containing (mM) 153 KCl, 10 HEPES, 1 MgCl_2_, 5 EGTA, and 1 MgATP (300 mOsm, pH 7.4) and clamped at −70 mV; the external solution contained (mM): 142 NaCl, 8 KCl, 10 HEPES, 10 Glucose, 6 MgCl_2_, 1 CaCl_2_ (325 mOsm, pH 7.4). Whole-cell recordings were acquired using an Axon MultiClamp 700B amplifier, filtered at 2 kHz, digitized at 10 kHz with Digidata 1440A, and recorded with Clampex 10.4 software (Molecular Devices, San Jose, CA, USA). Currents were elicited with 1 mM GABA application through a microcapillary using a gravity system. Peak current analysis was performed using ClampFit software 11.4.2.

Predictive structural modeling

We simulated the perturbations of six α1 mutations with an array of computational platforms: DynaMut2, mCSM, I-Mutant 2.0, and MUpro. The crystal structure of native human GABA_A_R (PDB ID: 9CRS) was retrieved from the RCSB Protein Data Bank. This structure was selected for its high resolution (2.9 Å) and complete β2-α1-β2-α1-γ2S subunit assembly. All seven point mutations were introduced in silico, and DynaMut2 was used to visualize tertiary structural changes and to predict ΔΔG and residue-level flexibility based on normal mode analysis.

Structural stability changes induced by variants were predicted using the α1 subunit from chain D of the receptor model. Because α1 subunits occupy distinct positions within the pentameric assembly, the local structural environment varies slightly between chains due to differences in inter-subunit interactions, which can lead to modest variation in predicted ΔΔG values. For consistency, all variants were modeled using chain D.

After independently analyzing each variant within each platform (Figure 1B), we averaged the predicted changes in Gibbs free energy (ΔΔG) across the platforms for each variant (Figure 1C). The modeling for M263T and benign variants as the control group was done using DynaMut2, mCSM, DUET, and SDM2.

Data analysis and statistics

Statistical data were stored using Microsoft Excel, analyzed and graphed using GraphPad Prism version 10. Data were found to be normally distributed, as in previous similar studies [46,47]. Values are displayed as means ± SEMs. For Western blot experiments, values per blot were typically normalized to loading controls, then to untreated WT samples within that blot, as specified in the figures. The significance was tested by a one-sample *t* test or one-way ANOVA, with Sidak’s multiple comparisons.

## 3. Results

### 3.1. GABRA1 Variants Destabilize α1 Subunit Structure and Disrupt Local Intermolecular Interactions

We began by performing in silico structural analysis of the mutations. GABA_A_Rs belong to a “cys-loop” superfamily of pentameric ligand-gated ion channels, and each subunit contains an extracellular N-terminal domain, four transmembrane (TM) domains with TM2 lining the channel pore, and an intracellular cytoplasmic loop between TM3 and TM4 [48]. The full length of the human α1 peptide is 456 amino acids. I148 and R214 residues are in the extracellular domain: I148 in the β-sheet region and R214 in the ligand-binding interface region. M263, T292, A322, and F325 residues are in TM domains: M263 in TM1, T292 in TM2, and A322 and F325 in TM3 (Figure 1A). We performed sequence alignment and evaluated sequence conservation for these residues, for which we included *GABRA1* sequences from fish, amphibians, birds, and mammals (Table 1). All six residues were highly conserved across the species, as were the neighboring residues, indicating their importance to GABA_A_R function.

We predicted structural implications of the variants using DynaMut2 with the human α1β2γ2S GABA_A_R structure (PDB: 9CRS), finding that most variants are particularly disruptive to native intermolecular interactions, including hydrogen bonds, polar contacts, and hydrophobic interactions (Figure 1B,C). R214 substitutions (R214C and R214H) alter charge and side-chain interactions, leading to rearrangement of the local environment and destabilized contacts. The A322D variant introduces a negatively charged residue into a hydrophobic region, resulting in unfavorable polarity interactions and steric strain. Similarly, the M263T variant introduces a polar residue into a hydrophobic transmembrane environment, disrupting local packing. The T292S substitution alters hydrogen bonding and local packing interactions despite being chemically conservative. In contrast, the F325L substitution preserves the hydrophobic character with only minor packing changes, consistent with its comparatively mild destabilizing effect.

We then estimated the change in protein stability for each variant using an array of computational platforms (Figure 1B,C). For most variants, the average predicted change in Gibbs free energy (ΔΔG) values were negative and exceeded the −0.5 kcal/mol threshold indicative of a destabilizing effect. These included I148F (−1.375 kcal/mol), R214C (−0.744 kcal/mol), R214H (−1.162 kcal/mol), T292S (−1.018 kcal/mol), and F325L (−0.671 kcal/mol). A322D (−0.475 kcal/mol) and M263T (−0.314 kcal/mol) showed comparatively smaller predicted energetic changes, though structural inspection still revealed disruption of the local physicochemical environment, including altered polarity, packing constraints, and unfavorable residue interactions. In contrast, several control variants with limited or no known disease association, including R214K (0.159 kcal/mol), F325Y (−0.194 kcal/mol), and P360L (0.317 kcal/mol), demonstrated consistently minimal energetic perturbation and preserved local interaction networks. It should be noted that in silico analyses of these three variants, which are reported in gnomAD or ClinVar, do not yield a general consensus from a predictive perspective.

Importantly, ΔΔG magnitude is not an absolute predictor of pathogenicity. The functional consequences of a variant depend not only on local thermodynamic stability, but also on the specific structural and functional context of the affected residue, including its role in receptor assembly, trafficking, channel gating, subunit interfaces, and conformational dynamics. Therefore, even variants associated with relatively modest predicted ΔΔG changes may still produce substantial physiological dysfunction when located within functionally sensitive regions. These findings support the use of structural modeling as a mechanistic framework complementary to molecular, functional, and clinical data, rather than as a standalone determinant of pathogenic severity. While in silico predictions offer a scalable approach for analyzing mutation effects, validating these findings in cell and animal models remains essential for drawing definitive conclusions.

### 3.2. Most GABRA1 Variants Show Reduced α1 Subunit in the HEK293T Cells Expressing the Variant α1β2γ2 Receptors Compared with the Wildtype HEK293T Cells

**Figure 2:**
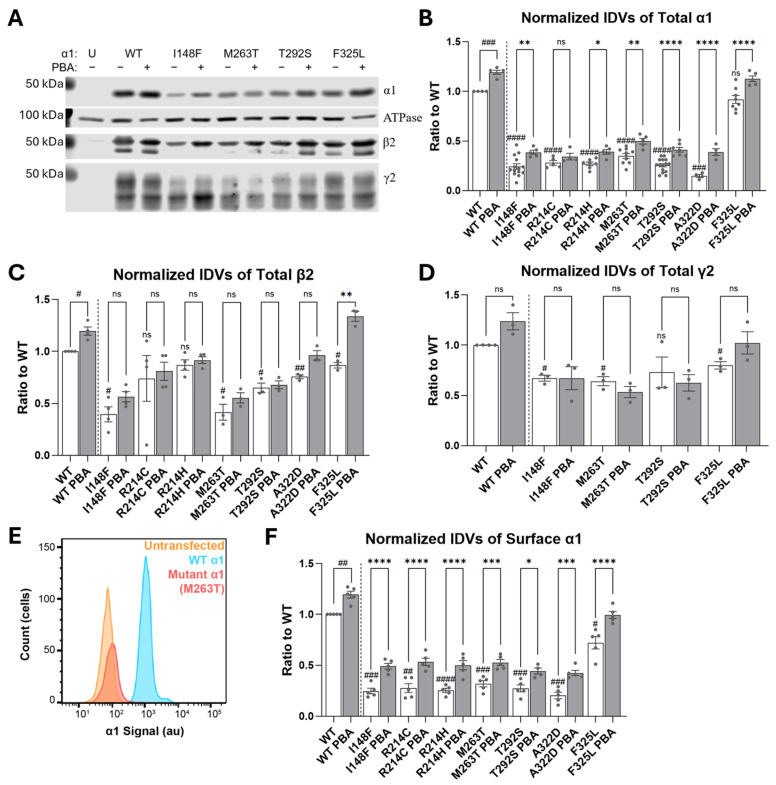
*GABRA1* variants show reduced α1 subunit expression that is partially rescued by PBA. (**A**) Western blot showing total expression of α1, β2, and γ2S subunits in HEK293T cells co-transfected with α1, β2, and γ2S cDNAs (1:1:1 ratio). For each condition, we varied whether the WT or variant α1 was transfected; for PBA treatment, 2 mM PBA was added 24 h prior to harvesting. ATPase was used as a loading control. (**B**) Quantification of total α1 protein levels normalized to WT. (**C**,**D**) Quantification of total γ2S and β2 protein; not every α1 blot included a γ2S and β2 co-stain, which is reflected in the smaller sample number for these blots. (**E**,**F**) Surface protein expression of α1 subunits quantified by flow cytometry using a monoclonal mouse anti-α1 antibody with Alexa-647 secondary. A representative example of flow cytometry results is shown in (**E**), with quantification normalized to untreated WT in (**F**). Data are presented as means ± SEMs; ####, ###, ##, # *p* < 0.0001, 0.001, 0.01, 0.05 by one-sample ratio *t* test vs. 1 (untreated WT); ****, ***, **, * *p* < 0.0001, 0.001, 0.01, 0.05 by one-way ANOVA with Sidak’s multiple comparisons; ns, not significant.

Next, we evaluated the impact of *GABRA1* variants on α1 subunit expression using homozygous expression in the heterologous HEK293T cell system. We chose α1β2γ2S as it is one of the most common synaptic GABA_A_R combinations and consistent with our structural modeling. We expressed either the WT or variant α1 co-transfected with β2 and γ2S subunits at a 1:1:1 ratio and examined α1 expression by Western blotting (Figure 2A). We observed that the I148F, R214C, R214H, M263T, T292S, and A322D variants all displayed a severe reduction in expression compared to WT (Figure 2B): decreases of 75.8% for I148F (#### *p* < 0.0001 vs. WT), 72.0% for R214C (#### *p* < 0.0001), 73.0% for R214H (#### *p* < 0.0001), and 74.5% for T292S (#### *p* < 0.0001); M263T was more mild (a 61.5% reduction, #### *p* < 0.0001), and A322D was most strongly affected (85.2% reduction, ### *p* < 0.0001). F325L was an exception and showed expression comparable to WT (9.1% decrease, *p* = 0.3181).

We also evaluated expression of the partnering β2 and γ2S subunits. For γ2, we were only able to evaluate expression for a subset of the variants I148F, M263T, T292S, and F325L. Compared to WT, γ2S expression was 32.9% lower for I148F (# *p* < 0.05), 36.0% lower for M263T (# *p* < 0.05), and 20.2% lower for F325L (# *p* < 0.05); for T292S, expression was 27.1% lower than WT, but this change was not significant (Figure 2C). β2 expression was 40.5% lower than WT for I148F (# *p* < 0.05), 58.5% lower for M263T (# *p* < 0.05), 35.0% lower for T292S (# *p* < 0.05), 24.4% lower for A322D (## *p* < 0.01), and 13.4% lower for F325L (# *p* < 0.05; Figure 2D). We did not detect any statistically significant changes in β2 expression for R214C or R214H.

### 3.3. PBA Improves α1 Expression for Most GABRA1 Variants

We next assessed whether α1 expression could be restored by treatment with PBA. We used a treatment protocol that we previously optimized to rescue expression of other *GABR* variants in HEK293T cells, which entails a 24 h treatment with 2 mM PBA prior to cell harvest. PBA treatment significantly increased α1 expression for most variants (Figure 2B): increases of 59.4% for I148F (** *p* < 0.01 vs. untreated), 44.4% for R214H (* *p* < 0.05 vs. untreated), 42.7% for M263T (** *p* < 0.01 vs. untreated), 61.5% for T292S (**** *p* < 0.0001 vs. untreated), and 163% for A322D (**** *p* < 0.0001 vs. untreated) were observed. For R214C, treated expression values were 22.4% greater than untreated values, but this increase was not statistically significant (*p* = 0.8350). A 19.2% increase was also observed for WT α1 (### *p* < 0.01 vs. untreated WT), and a 19.1% increase was observed for F325L (**** *p* < 0.0001 vs. untreated), which did not show a baseline reduction in expression. However, PBA treatment did not fully restore the expression of α1 variants to WT levels; despite the improvements, all α1 variants that showed reduced expression at baseline remained at 34–50% of baseline WT levels following PBA treatment.

We detected no changes in γ2S for PBA vs. untreated samples of WT, I148F, M263T, T292S, and F325L variants (Figure 2C), nor in β2 for I148F, R214C, R214H, M263T, and T292S (Figure 2D). β2 expression increased with PBA treatment by 19.5% for WT (# *p* < 0.05 vs. untreated WT) and by 54.5% for F325L (** *p* < 0.01 vs. untreated). Notably, for WT and several variants, we noticed an upward trend in expression values, and it is possible that a small percent increase could be resolved with higher statistical power.

### 3.4. PBA Improves Surface Expression in α1β2γ2-Expressing HEK293T Cells

Next, we investigated α1 surface expression of the α1 variants using flow cytometry. Variants that showed reduced total α1 expression also showed a comparable reduction in α1 surface expression (Figure 2E,F): a 75.2% reduction compared to WT for I148F (### *p* < 0.001), 72.2% for R214C (## *p* < 0.01), 74.6% for R214H (#### *p* < 0.0001), 68.0% for M263T (### *p* < 0.001), 72.6% for T292S (### *p* < 0.001), and 79.6% for A322D (### *p* < 0.001). The F325L variant showed a moderate reduction in surface α1 expression (28.0% less than WT, # *p* < 0.05), though total expression was not significantly reduced.

PBA treatment improved α1 surface expression for WT and all of the variants (Figure 2F): 19.4% increase vs. untreated for WT (### *p* < 0.001), 97.6% for I148F (**** *p* < 0.0001), 91.4% for R214C (**** *p* < 0.0001), 96.9% for R214H (* *p* < 0.05), 64.4% for M263T (** *p* < 0.01), 61.5% for T292S (**** *p* < 0.05), 108% for A322D (**** *p* < 0.001), and 38.1% for F325L (**** *p* < 0.0001).

**Figure 3:**
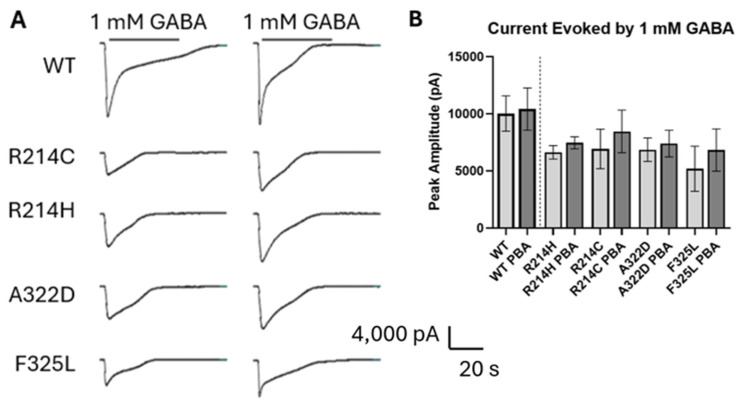
Lower peak GABA current in HEK293T cells expressing α1-variant-containing GABA_A_Rs. Current from cells expressing the WT or variant receptors was elicited by 1 mM GABA application in HEK293T cells expressing α1β2γ2S at a 1:1:0.5 ratio with and without PBA treatment. (**A**) Sample traces. (**B**) Quantification of peak current amplitude for all variants. *n* = 10 cells for WT, 8 for WT + PBA, 6 for R214H, 4 for R214H + PBA, 4 for R214C, 4 for R214C + PBA, 5 for A322D, 6 for A322D + PBA, 4 for F325L, and 5 for F325L + PBA. Data shown as means ± SEMs.

Additionally, we performed an electrophysiologic evaluation of whole-cell currents elicited by 1 mM GABA application (Figure 3). We evaluated WT and R214C, R214H, A322D, and F325L variants with and without PBA in HEK293T cells. Compared to WT, all variants showed a lower average current amplitude that trended upwards with PBA treatment. However, these trends were not statistically significant, in part due to the high variance and limited scope of this experiment.

### 3.5. Total and WT α1 Expression with “Heterozygous” α1α1^HA^β2γ2S Expression

**Figure 4:**
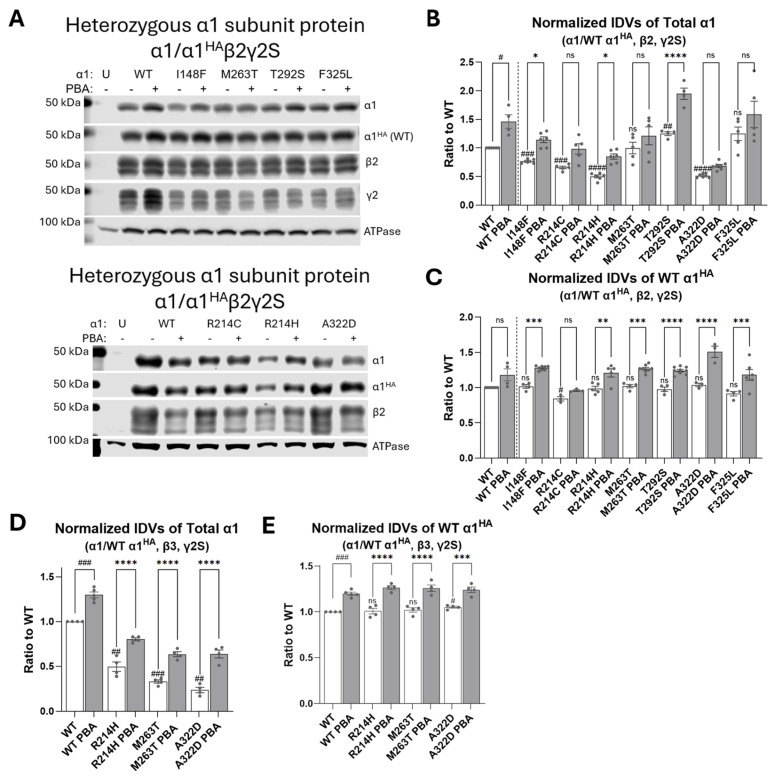
PBA restores total and WT α1^HA^ subunit expression in “heterozygous” GABRA1 variant conditions in α1β2γ2S and α1β3γ2S receptors. (**A**) Western blot showing total expression of α1, HA-tagged α1^HA^, β2, and γ2S subunits in HEK293T cells co-transfected with α1, α1^HA^ β2, and γ2S cDNAs (0.5:0.5:1:1 ratio). For each condition, we varied whether the WT or variant α1 was transfected, while α1^HA^ was always kept as the WT; for PBA treatment, 2mM PBA was added 24 h prior to harvesting. ATPase was used as a loading control. For a limited number of conditions, we included additional co-expression with the β3 subunit, sample blots for which are shown in Figure S2. (**B**,**C**) Quantification of total α1 (**B**) and WT α1^HA^ (**C**) protein levels normalized to the untreated WT condition (per blot) in the “heterozygous” α1/α1^HA^β2γ2S conditions. β2 and γ2S quantification is shown in Figure S1. (**D**,**E**) Quantification of total α1 (**B**) and WT α1^HA^ (**C**) protein levels normalized to untreated WT condition (per blot) in the subset of variants co-expressed in “heterozygous” α1/α1^HA^β3γ2S conditions. Data are presented as means ± SEMs (*n* = 4–9; plotted as dot plots in each graph). Statistical analysis was performed using one-way ANOVA followed by Sidak’s multiple-comparisons test. Data are presented as means ± SEMs; ####, ###, ##, # *p* < 0.0001, 0.001, 0.01, 0.05 by one-sample ratio *t* test vs. 1 (untreated WT); ****, ***, **, * *p* < 0.0001, 0.001, 0.01, 0.05 by one-way ANOVA with Sidak’s multiple comparisons; ns, not significant.

Next, we sought to recapitulate the heterozygous state of the patients by co-expressing α1 variants/WT α1 control together with an HA-tag labeled WT α1 (α1^HA^) and β2 γ2S partnering subunits at a 0.5:0.5:1:1 ratio, respectively. Under these conditions, the total α1 expression was significantly reduced compared to WT: reductions of 23.6% for I148F (### *p* < 0.001), 34.4% for R214C (### *p* < 0.001), 50.0% for R214H (#### *p* < 0.0001), and 48.5% for A322D were observed (#### *p* < 0.0001; Figure 4A,B). For M263T, T292S, and F325L, total α1 expression was not reduced. In contrast, the WT α1^HA^ allele was expressed at comparable levels in both WT and α1 variant conditions with the exception of R214C, which showed a 15.9% reduction (# *p* < 0.001 vs. WT; Figure 4C).

### 3.6. PBA Treatment Increased Total and WT α1 Expression with “Heterozygous” α1α1^HA^β2γ2S

Total α1 expression values for the PBA treatment condition were greater than for the untreated condition in all cases (Figure 4B), but this increase was only statistically significant for WT (46.2% increase; # *p* < 0.05 vs. untreated), I148F (49.7% increase; * *p* < 0.05 vs. untreated), R214H (69.9% increase; **** *p* < 0.0001 vs. untreated), and T292S (55.9% increase; **** *p* < 0.0001 vs. untreated). For I148F and R214H, expression values with treatment surpassed WT baseline expression. Expression values of the WT allele α1^HA^ were also greater than for the untreated condition in all cases (Figure 4C), and this increase was significant for all variants except R214C and WT: 25.8% for I148F (**** *p* < 0.0001 vs. untreated), 22.6% for R214H (** *p* < 0.001 vs. untreated), 24.3% for M263T (*** *p* < 0.0001 vs. untreated), 27.1% for T292S (**** *p* < 0.0001 vs. untreated), and for 29.3% F325L (**** *p* < 0.0001 vs. untreated).

### 3.7. Expression of Partnering β2 and γ2S Subunits with “Heterozygous” α1α1^HA^β2γ2S

We evaluated γ2S expression for the I148F, M263T, T292S, and F325L variants and observed lower expression values compared to WT for all of them; however, only the 26.5% decrease for M263T was significant (# *p* < 0.05). PBA treatment increased γ2S expression in the WT condition by 29.0% (**** *p* < 0.0001 vs. untreated); however, we did not observe a similar increase in the variants: I148F showed a moderate 16.7% increase (* *p* < 0.05), while M263T, T292S, and F325L showed no change, nor was there a trend in the values.

Expression of β2 showed no significant changes for the variants, though the values across the variants were typically slightly lower than for WT (Figure S1). Similarly, PBA treatment showed no significant changes in β2 expression across the groups, though values tended to be larger than those for the untreated condition.

Our sample size and statistical power were lower for the γ2S and β2 expression experiments, and it is possible that significant changes in the direction of the indicated trends would be identified with an expanded sample size.

### 3.8. Stronger Decrease in Total α1 Expression for M263T and A322D with β3 Partnering Subunit

Next, we assessed whether subunit composition influences the stability of the variant-containing GABA_A_Rs by performing parallel experiments with the β3 partnering subunit in α1/α1^HA^β3γ2S co-expression conditions (Figure 4D,E), which is another highly common synaptic subunit combination in the brain. For these experiments, we included only the R214H, M263T, and A322D variants, all of which showed a strong decrease in total α1 expression compared to the untreated WT: decreases of 50.5% for R214H (## *p* < 0.01 vs. untreated WT), 66.7% for M263T (### *p* < 0.001), and 76.2% for A322D (## *p* < 0.01) were observed. While the decrease for R214H was comparable to those for the α1/α1^HA^β2γ2S conditions, it was stronger for A322D and much stronger for M263T, which did not show any reduction in the α1/α1^HA^β2γ2S conditions. Expression of the WT α1^HA^ allele was comparable to WT across these conditions and did not indicate significant dominant negative effects or compensation.

### 3.9. PBA Treatment Showed a Stronger Increase in Total and WT α1 Expression with “Heterozygous” α1/α1^HA^β3γ2

Treatment with PBA strongly increased α1 expression for these variants (Figure 3B): 62.2% for R214H (**** *p* < 0.0001 vs. untreated), 91.1% for M263T (**** *p* < 0.0001 vs. untreated), 168.4% for A322D (**** *p* < 0.0001 vs. untreated), and 29.8% for WT (### *p* < 0.001). Again, this was on par with the α1/α1^HA^β2γ2S conditions for R214H, but a much stronger increase was shown for A322D and M263T. PBA also increased expression of the WT α1^HA^ allele similarly across the three variants (Figure 4E): 25.5% for R214H (**** *p* < 0.0001 vs. untreated), 23.3% for M263T (**** *p* < 0.0001 vs. untreated), 18.1% for A322D (*** *p* < 0.001 vs. untreated), and 19.8% for WT (### *p* < 0.001). In summary, the co-expressed subunits seemed to make a palpable difference to the stability and expression of variant-containing GABA_A_Rs.

In addition to the variants that are the focus of this study, we also evaluated I148P and M263K that occur at the same peptide locations as I148F and M263T, respectively (Figure S2). Compared to WT in α1/α1^HA^β2γ2S conditions, α1 expression was 63.3% lower for I148P (## *p* < 0.01 vs. untreated WT) and 77.0% lower for M263K (## *p* < 0.01 vs. untreated WT), and it increased with PBA treatment by 74.4% for I148P (**** *p* < 0.0001 vs. untreated) and 165.2% for M263K (**** *p* < 0.0001 vs. untreated). Expression of the WT α1^HA^ allele was comparable for I148P to WT and showed a comparable response to PBA treatment (22.1% increase vs. untreated; **** *p* < 0.0001). On the other hand, M263K demonstrated the strongest dominant negative effect we observed among the variants, showing a strong decrease in WT α1^HA^ expression (66.2% reduction vs. WT; ## *p* < 0.01), and PBA treatment was highly effective in treating this phenotype (212.0% increase vs. untreated; **** *p* < 0.0001).

Together, these findings demonstrate that *GABRA1* variants impair total α1 subunit expression under heterozygous conditions, while WT α1 expression is relatively preserved (except M263K). PBA treatment partially restores total α1 levels and increases WT α1 expression across both β2γ2S and β3γ2S receptor assemblies. However, the magnitude of this increase is often smaller than the increase seen in total α1 expression, which we also observed with α1^variant^β2γ2S. Therefore, the PBA rescue effect appears to involve enhancing the expression of both the WT and variant α1.

### 3.10. PBA Increases α1 Expression in a Region-Specific Manner in the Gabra1^+/A322D^ Mouse Brain

Next, we sought to determine whether PBA treatment can improve α1 expression in vivo. To do this, we used the *Gabra1*^+/A322D^ mice that have previously been shown to express α1 at about half the WT levels [40]. We followed a 7-day treatment regimen with vehicle or 100 mg/kg/day PBA via intraperitoneal (IP) injection based on our prior studies in *Slc6a1*^+/S295L^ and *Gabrg2*^+/Q390X^ mice [21,22,23]. Then, we collected the mouse brain tissue and quantified α1, β2, and γ2 expression by Western blotting on tissue lysates from the cortex, cerebellum, hippocampus, and thalamus normalized to ATPase (Figure 4A).

**Figure 5:**
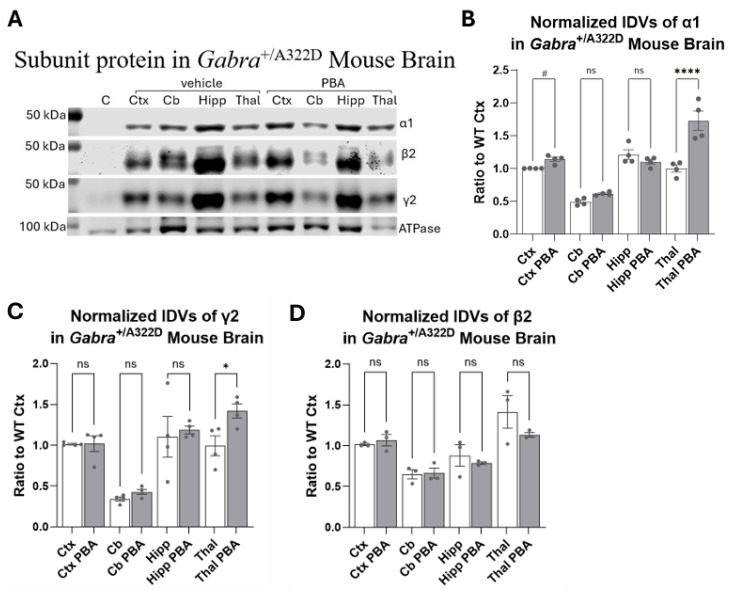
PBA increases α1 subunit expression in a region-specific manner in the brains of *Gabra1*^A322D/−^ mice. (**A**) Western blots showing α1, β2, and γ2 subunit expression in tissue lysates from cortex (Ctx), cerebellum (Cb), hippocampus (Hipp), and thalamus (Thal) from *Gabra1*^A322D/−^ mice treated with vehicle or 4-phenylbutyrate (PBA); ATPase was used as a loading control. (**B**–**D**) Quantification of (**B**) α1, (**C**) β2, and (**D**) γ2 protein levels normalized to the vehicle-treated *Gabra1*^A322D/−^ mice. Data are presented as means ± SEMs; # *p* < 0.05 by one-sample ratio *t* test vs. 1 (untreated Ctx); ****, * *p* < 0.0001, 0.05 by one-way ANOVA with Sidak’s multiple comparisons; ns, not significant. *n* = 4 mice for treated and untreated groups (8 total).

We found that PBA treatment increased α1 expression in the *Gabra1*^+/A322D^ cortex by 14.0% (# *p* < 0.05) and the thalamus by 72.8% (**** *p* < 0.0001) but that it did not affect expression in other regions (Figure 5A,B). We observed a matching 42.0% increase in γ2 expression in the thalamus (**** *p* < 0.0001; Figure 5C), but not in other regions. We did not detect changes in β2 expression in any of the four regions (Figure 5D).

### 3.11. PBA Mitigated Seizures in Patient Carrying GABRA1(R214C) Variant

Based on our preclinical data, the phase I/II trial (NCT04937062) was expanded to the *GABRA1* variants and others, which is still ongoing. Here, we report a child heterozygous for the *GABRA1*(R214C) variant. The child was diagnosed with DEE, attention hyperactivity deficit disorder (ADHD) and autism spectrum disorder by age 7 (Table 2, Pre-Ravicti treatment). The initial electroencephalogram (EEG) reading at rest was normal despite a history of bilateral tonic clonic seizures. Seizures progressively worsened over time with mixed seizure semiology, including absence, focal and bilateral tonic clonic seizures, despite multiple antiseizure medications. Following Ravicti addition, the child achieved seizure freedom for 245 days with improved emotional regulation and social interaction (Table 2, Post-Ravicti treatment).

## 4. Discussion

In this study, we evaluated the pathophysiology and potential of PBA, known as a chemical chaperone and histone deacetylase inhibitor, for seven *GABRA1* variants associated with DEEs, including I148F, R214C, R214H, M253T, T292S, A322D, and F325L. Using in silico modeling, we identified that the variants occur in highly conserved residues and act to destabilize the α1 subunit to varying degrees. The variants demonstrated reduced total (except F325L) and surface expression in HEK293T cells. This is consistent with in silico modeling that predicts reduced protein stability in the variant proteins of α1 subunits. For half of the variants, the reduction in total α1 expression persisted in “heterozygous” co-expression with WT α1 and α1^HA^, though dominant negative effects on the WT subunit were not observed. PBA treatment improved both total (except R214C) and surface expression of the variants (including R214C) and the WT α1 subunit in the control condition and with “heterozygous” co-expression with variants. These findings suggest a shared folding and trafficking pathology of varying severity for these variants and showcase that PBA is an effective treatment that improves expression of both mutant and WT α1 subunits at the cell surface. The increase in surface α1 subunits is likely correlated with the increase in GABA_A_R function, thus mitigating disease phenotypes.

At the structural level, computational modeling predicted that all variants would exhibit destabilizing effects on the α1 subunit protein, consistent with disruption of intermolecular interactions within the N-terminal domain and transmembrane helices. Such perturbations are expected to impair subunit folding and assembly, thereby compromising receptor maturation. This is consistent with the negative ΔΔG predicted change in stability values across multiple computational platforms. The ΔΔG values also matched our predictions based on structural visualization of the mutations and the highly conserved status of the residues. However, the change in ΔΔG magnitude was a weak predictor for the magnitude of decrease in subunit expression in some variants. For example, the F325L variant showed the least change in α1 expression among the variants, which matched the smaller ΔΔG magnitude relative to other variants. Contrary to this trend, however, the A322D variant exhibited the strongest decrease in α1 expression but displayed the smallest predicted ΔΔG magnitude. Nevertheless, in silico modeling, combined with biochemical experiments, is a valuable tool for large-scale protein stability predictions.

We found that most variants showed a similarly strong decrease in α1 expression when expressed as the only α subunit but showed differences when co-expressed with WT α1^HA^. This was observed in our previous study and suggests that the mutant α1 subunit is in a weak position when competing with the WT allele for protein folding [41]. In the α1β2γ2S conditions, both total and surface α1 expression decreased in the 61–85% range compared to the WT control. F325L was the only exception and showed an increase in surface expression. However, in “heterozygous” α1/α1^HA^β2γ2S conditions, only the variants I148F, R214C, R214H, and A322D showed decreased α1 expression, while the M263T and T292S variants did not. This was unlikely to happen due to compensatory WT α1 expression alone because WT α1^HA^ expression levels were comparable between the WT and most variants. One possible explanation is that the WT α1^HA^ subunit stabilized the formation of GABA_A_Rs containing a single WT and a single variant α1 subunit, which has previously been reported for specific configurations with the A322D variant [29]. This stabilizing effect would differ by variant and is particularly plausible in the case of M263T based on the milder expression phenotype under α1β2γ2S conditions. This could also explain why the M263T variant showed a significant decrease in α1 expression in the α1/α1^HA^β3γ2S condition—the substitution of the β3 for the β2 subunit appeared to form a less stable configuration with the variant (Figure 4). This could be related to the fact that the β3 subunit can form homo-pentamers, thus further weakening the mutant α1 subunit during receptor assembly.

Because of the critical role of α1 subunit in the pentameric receptor assembly, the expression of the partnering subunits β2 and γ2S was also decreased when co-expressed with the mutant α1 variants. This was particularly consistent for γ2S, including both α1β2γ2S and α1/α1^HA^β2γ2S conditions. This is likely to be due to the fact that the stoichiometry of the pentameric receptor can be either that of the αβ or the αβγ2 receptor, and the β subunit can substitute the α subunit in the receptor while the γ2 subunit cannot. The reduction in the expression of the partnering β2/3 and γ2S subunits is likely due to the reduced availability of the partnering α1 subunit.

Compared to existing expression studies, our findings match some of the reports but also show some differences. Our findings match the extensive prior A322D characterization including reduced total and surface α1 expression, a mild dominant negative effect, and a strong reduction in GABA_A_R current magnitude [28,29,30] and the initial characterization of R214C showing reduced receptor current with decreased α1 membrane and total expression [34]. Our findings also match the reduced GABA_A_R current magnitude reported by Hernandez et al. for R214C and R214H. Additionally, we observed reduced α1 expression in addition to a previously reported gating defect [33]. Our findings for T292S differed from a previous report that the mutation causes gain in GABA_A_R function associated with increased ligand sensitivity but normal total and surface expression [37]. While we did not find evidence contrary to changes in GABA_A_R sensitivity, T292S showed a significant decrease in both total and surface α1 expression (Figure 2) and only showed normal levels when co-expressed with WT α1^HA^. We are unsure as to the source of the discrepancy in our expression results, as the aforementioned studies [33,37] used similar methods, including the HEK293T cell system and the β2 and γ2S partnering subunits. For the I148F, F325L, and M253T variants, our study is the first functional characterization.

Treatment with PBA caused a significant increase in the expression of α1 protein, including WT and mutant variants. For WT α1, we observed higher total and surface α1 expression in the WT control α1β2γ2S conditions and α1/α1^HA^β2γ2S conditions. Additionally, WT α1^HA^ was expressed at higher levels in “heterozygous” α1/α1^HA^β2γ2S co-expression with most α1 variants, and the magnitude of this increase was generally comparable to that of the WT α1 control. Therefore, it seems that PBA increased α1 expression regardless of the presence of the mutant protein, which is beneficial in case of a haploinsufficiency disorder. On the other hand, PBA also increased α1 expression in cases when only the variant α1 was transfected, indicating that the variant α1 proteins were also stabilized and increased in expression. Therefore, increased total α1 expression in “heterozygous” conditions likely reflects improvements in both the WT and mutant α1 protein expression as well as general stabilization of the GABA_A_R pentamer.

PBA treatment increased both the total and surface α1 subunit protein expression across variants. However, the magnitude of increase in the total and surface expression may vary. This is likely due to the fact that overexpression of the recombinant receptors may overwhelm the ER protein quality control machinery and make the protein trafficking environment less conducive. Nevertheless, PBA increased the surface GABA_A_Rs, suggesting an increase in receptor channel function across variants. This is consistent with the reduction in seizures and neurobehavioral abnormalities.

We showed that PBA treatment also improved α1 expression in vivo in *Gabra1*^+/A322D^ mice. *Gabra1*^+/A322D^ mice express roughly half the WT levels of α1 [40], and we observed a strong 72.8% increase in thalamic and a moderate 14.0% increase in cortical α1 expression with the PBA treatment. However, we did not observe an increase in the other regions, though α1 is expressed widely throughout the brain, with high expression in all of the studied regions [49]. This indicates a regional effect and reflects increased in vivo complexity compared to in vitro reductionism, including the presence of many other partnering subunits. A rescue in these regions may be better evaluated by surface expression and synaptic function. Nonetheless, we found these results promising and believe that there is high potential in evaluating whether PBA treatment can reduce the seizure burden in *Gabra1*^+/A322D^ mice [40] as it has for other models [21,22]. Importantly, PBA (Ravicti) mitigated seizures and neurobehavioral abnormalities in the patient carrying *GABRA1*(R214C) (Table 2). This suggests that the preclinical data in our study are predictive of efficacy in human patients with *GABRA1* mutations.

**Figure 6:**
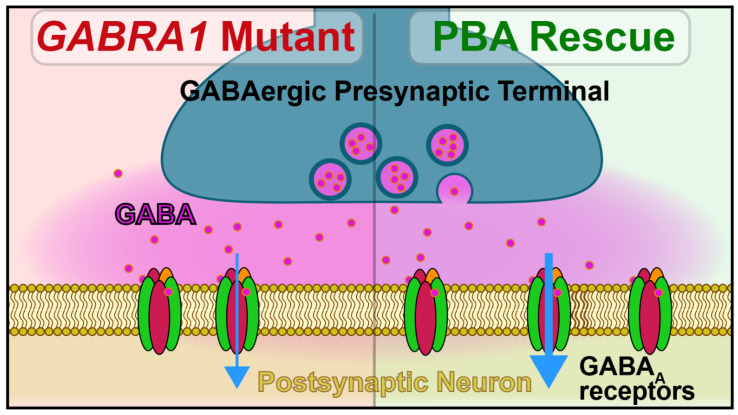
Our proposed model of a shared pathomechanism for many *GABRA1* variant-mediated disorders. This is based on this study and previous work in similar disorders. *GABRA1* variants affect GABA_A_R proteostasis, reducing the receptor number at the synapse and weaking synaptic responses. PBA stabilizes proteostasis and increases GABA_A_R subunit expression, improving receptor number at the synapse and restoring synaptic response, thus mitigating seizures and improving neurobehavioral phenotypes.

Together, our findings support a model of a common proteostasis pathology for *GABRA1* variants that is highly responsive to PBA treatment (Figure 6). In our prior work, we demonstrated this concept in DEEs associated with *SLC6A1* and *GABRG2* variants. Mutations destabilize the variant proteins, causing defective folding, inefficient assembly, and reduced trafficking. Misfolded or incompletely assembled subunits are likely to be retained within the endoplasmic reticulum and subjected to degradation pathways, ultimately reducing receptor surface availability [3,47,50]. The significant and consistent reduction in α1 expression and the restorative benefits of PBA treatment that we observed for most *GABRA1* variants are evidence for this model and strengthen the rationale of clinical trial development for *GABRA1*-variant-associated DEEs. Moreover, more targeted future evaluations would be beneficial in further testing this model, including a direct evaluation of variant α1 subunit trafficking in the endoplasmic reticulum; interactions between variant α1 subunits and endoplasmic reticulum proteostasis components, including chaperones BiP and calnexin; and thorough electrophysiologic evaluation of the variants and PBA therapeutic effects on seizure burden in mouse models, including *Gabra1*^+/A322D^ mice.

## Figures and Tables

**Table 1:** Alignment and putative mutation effects at GABRA1 patient residues across vertebrates.

Alignment at Variant Residues			
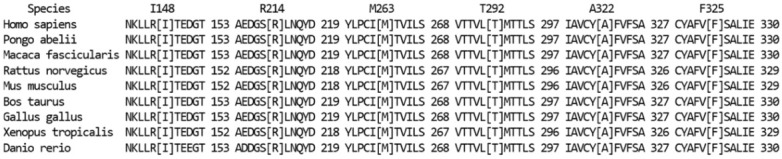			
**Variant**	**Domain**	**Expected biochemical impact**	**Residue conservation**
**I148F**	Extracellular domain (β-sheet region)	Hydrophobic → aromatic substitution; may alter local packing within extracellular β-sheet with modest structural perturbation	**Highly conserved hydrophobic residue** across all examined species (Ile preserved)
**R214C**	Extracellular domain (ligand-binding interface region)	Loss of positive charge and introduction of cysteine; potential disruption of electrostatic interactions and aberrant disulfide formation	**Highly conserved basic residue** (Arg invariant across species)
**R214H**	Extracellular domain (ligand-binding interface region)	Partial retention of positive charge; may weaken electrostatic interactions compared to Arg	**Highly conserved basic residue** (Arg invariant; His not observed)
**M263T**	Extracellular–TM1 interface	Hydrophobic → polar substitution; may disrupt packing at the extracellular–transmembrane transition and affect gating	**Highly conserved hydrophobic residue** (Met invariant across species)
**T292S**	TM2 (pore-lining helix)	Conservative polar substitution; minimal structural disruption expected, possible subtle effects on pore properties	**Highly conserved polar residue** (Thr invariant in alignment)
**A322D**	TM3	Small hydrophobic → negatively charged substitution; likely destabilizes transmembrane helix and local environment	**Highly conserved small hydrophobic residue** (Ala invariant across species)

For alignment, curated protein sequences were preferred, *GABRA1* orthologs were obtained from UniProt (reviewed Swiss-Prot entries) for *Homo sapiens* (P14867), *Bos taurus* (P08219), *Mus musculus* (P62812), *Rattus norvegicus* (P62813), *Gallus gallus* (P19150), *Macaca fascicularis* (Q4R534), and *Pongo abelii* (Q5R6B2), and from NCBI RefSeq for *Danio rerio* (NP_001070794.1) and *Xenopus tropicalis* (XP_031754594.1 as no curated entry for amphibians was identified). Sequences were aligned using MAFFT (https://www.genome.jp/tools-bin/mafft) (accessed on 10 April 2026) with the L-INS-i algorithm. Residue positions shown correspond to species-specific numbering. Variant residues are indicated in brackets.

**Table 2:** Clinical summary of a patient with *GABRA1*-associated developmental and epileptic encephalopathy before and after Ravicti treatment.

	Pre-Ravicti Treatment	Post-Ravicti Treatment
**Development**	Born at 37 weeks; required 5-day NICU stay for respiratory/feeding support and hyperbilirubinemia treatment. Normal early developmental milestones at 6 months. At 13 months, cruising but not yet walking and speaking 2–3 words.	Improved cognitive and behavioral function, including enhanced sequencing, cause-effect understanding, communication, attention, patience, and conversational skills. Learned birthday, letters, spelling, and ability to write own name. Reduced frustration and fewer behavioral meltdowns reported by family and school.
**Diagnosis**	Developmental and epileptic encephalopathy associated with *GABRA1* c.640C > T (p.Arg214Cys).	Same diagnosis maintained.
**Seizure Phenotype**	Seizure onset at 6 months with daily limb jerks (1–3 episodes/day). By 13 months, developed bilateral tonic-clonic seizures. Drug-resistant epilepsy with mixed seizure types including absence, focal, and bilateral tonic-clonic seizures. Weekly motor seizures, monthly bilateral tonic-clonic seizures, and multiple daily non-motor seizures.	Following Ravicti initiation, seizure free for approximately 245 days. Breakthrough focal seizure cluster occurred during viral illness, followed by staring spells and bilateral tonic-clonic seizures; one later seizure possibly associated with missed medication doses. Subsequently remained seizure-free for an additional 4 months with continued cognitive improvement.
**Neurodevelopmental/Behavioral Features**	Diagnosed with ADHD and autism spectrum disorder by age 7; receiving therapy services.	Improved emotional regulation, reduced frustration, improved social interaction, and enhanced school performance.
**EEG**	Brain MRI was normal. Initial EEG at approximately 13 months of age was normal despite development of bilateral tonic-clonic seizures. Clinical seizures progressively worsened over time with mixed seizure semiology including absence, focal, and bilateral tonic-clonic seizures despite multiple antiseizure medications.	Follow-up EEG (2025) demonstrated spike and polyspike-and-slow-wave discharges activated by photic stimulation and hyperventilation, with focal right hemispheric dysfunction.
**Treatments**	Treated with levetiracetam, oxcarbazepine, lamotrigine, topiramate, eslicarbazepine, clonazepam, and valproic acid with persistent drug-resistant seizures. Verapamil (10 mg) was trialed but discontinued after development of atrial fibrillation after 4 days.	Ravicti initiated at 2.5 mL three times daily and later weight-adjusted to 14 mL/day. Topiramate gradually weaned following prolonged seizure reduction.
**Family History**	Maternal uncle with childhood seizures, mother with migraines, and sister with ADHD.	No change.

## Data Availability

Data are available upon request from the corresponding author.

## Supplementary Materials

The following supporting information can be downloaded at: https://www.mdpi.com/article/10.3390/cells15151327/s1, Figure S1. Expression of β2 and γ2 partnering subunits in “heterozygous” co-expression conditions with and without PBA treatment. Figure S2. Total α1 and WT α1HA expression with β3 and γ2 partnering subunits in “heterozygous” co-expression conditions with and without PBA treatment (expanded from Figure 4D,E).
