# Supplementary material for: 4-Phenylbutyrate Rescue in GABRA1 Variants Associated with Developmental Epileptic Encephalopathies: From Cell and Mouse Models to Humans
Source: Cells. 2026 Jul 24;15(15):1327. doi: 10.3390/cells15151327 (PMC13465587; doi:10.3390/cells15151327)
Supplement: Supplementary file 1 [file cells-15-01327-s001.zip › cells-4310591-supplementary.pdf]

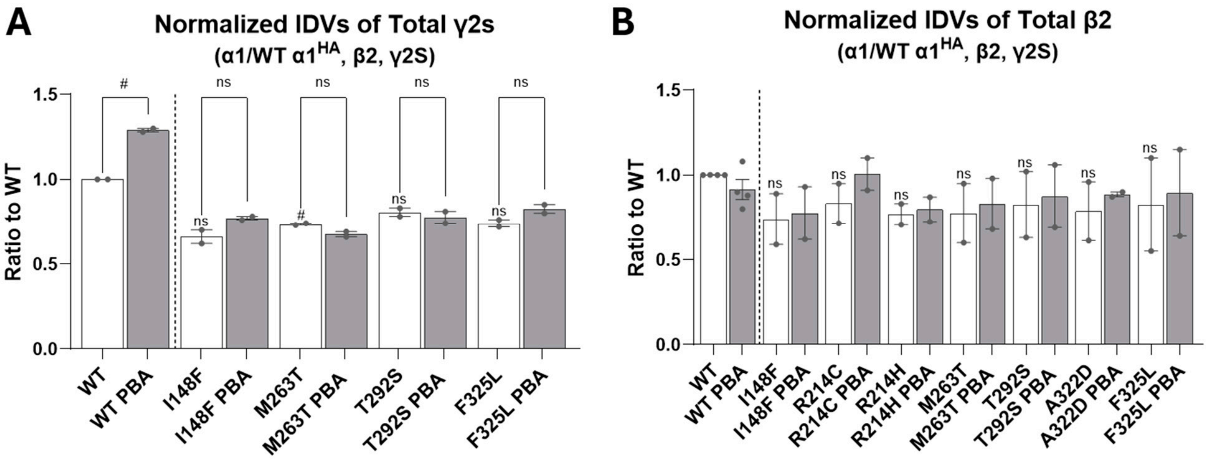

Figure S1. Expression of β2 and γ2 partnering subunits in "heterozygous" co-expression conditions with and without PBA treatment. Graphs display (A) γ2 and (B) β2 expression levels measured by Western blot in HEK293T cells co-expressing WT or variant α1, WT $\alpha1^{HA}$, β2, and γ2S partnering subunits at a 0.5:0.5:1:1 ratio. These data match Western blot images and α1 quantification shown in Figure 3A-C. Graphs show mean ± SEM with one-way ANOVA with Sidak multiple comparisons; ###, ##P < 0.001, 0.01 vs untreated WT; ****,*P < 0.0001, 0.05 vs respective untreated condition as indicated by brackets.

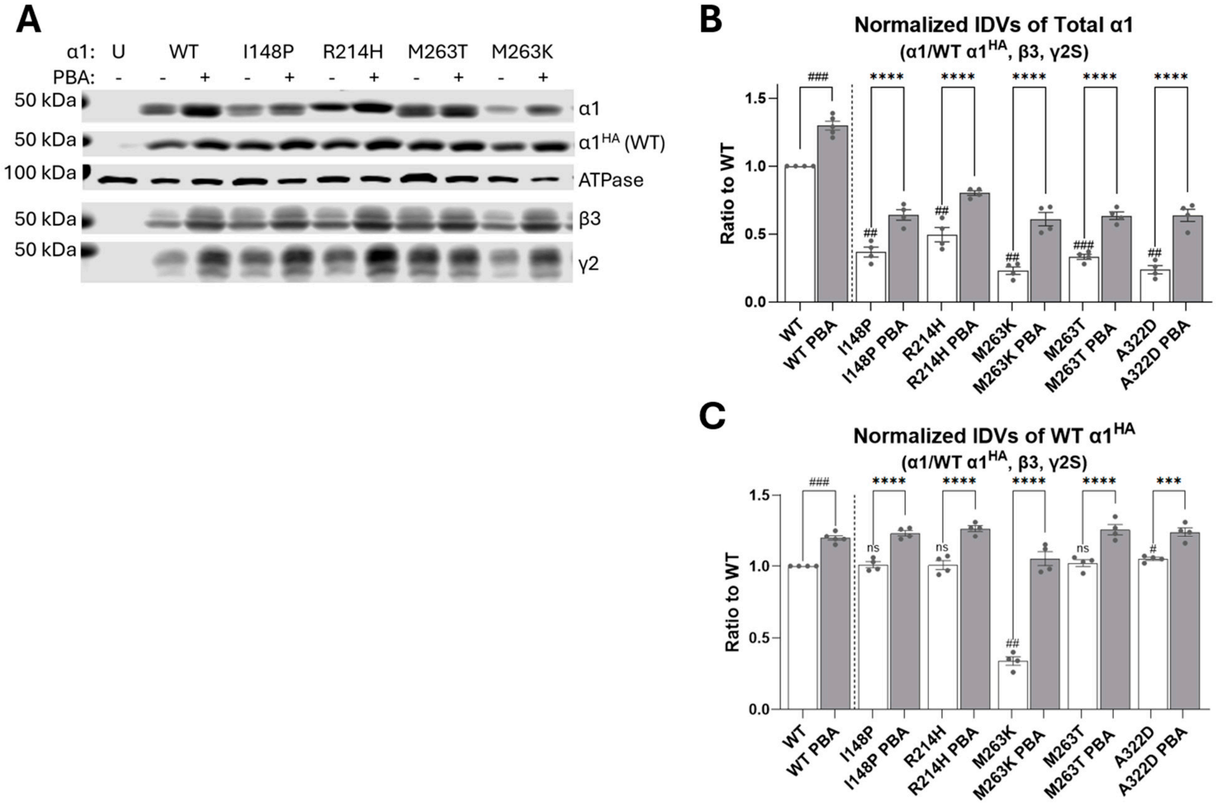

Figure S2. Total α1 and WT α1HA expression with β3 and γ2 partnering subunits in "heterozygous" co-expression conditions with and without PBA treatment (expanded from Figure 4D-E). (A) Sample Western blot showing the expressed α1, α1HA, β3, and γ2S subunits for some of the subunit conditions. (B) Quantification of total α1 expression. (C) Quantification of total WT α1HA expression. Note that only the R214H, M263T, and A322D variants out of the main dataset in this study were tested. Additional variants I148P and M263K that share the residue location with I148F and M263T variants investigated in this study were included. Graphs show mean ± SEM. Significance of untreated variants and treated WT tested by one sample ratio t test vs 1 (untreated WT); ####, ###P < 0.0001, 0.001; pairwise comparisons of treated vs untreated variants tested with one-way ANOVA with Sidak's multiple comparisons; ****P < 0.0001.
